# Vestibular evoked myogenic potentials (VEMP) captured in the forearm flexor muscles: a study of its feasibility and reference ranges

**DOI:** 10.6061/clinics/2020/e2020

**Published:** 2020-11-02

**Authors:** Maria Clara Motta Barbosa Valente, Aline Tenório Lins Carnaúba, Janise Dal Pai, Kelly Cristina Lira de Andrade, Pedro de Lemos Menezes

**Affiliations:** I Universidade Estadual de Ciencias da Saude de Alagoas (UNCISAL), Alagoas, SE, BR; II Universidade Estadual de Ciencias da Saude de Alagoas (UNCISAL) e Centro Universitario (CESMAC), Alagoas, SE, BR; III Universidade Federal de Sao Paulo (UNIFESP), Sao Paulo, SP, BR

**Keywords:** Vestibular Evoked Myogenic Potentials, Diagnosis, Vestibular Nerves

## Abstract

**OBJECTIVE:**

To determine the central tendency measures and variability of vestibular evoked myogenic potential (VEMP) with regard to the latency and wave amplitude when potentials are captured from the flexor muscles of the forearm.

**METHODS:**

Ten adult volunteers with normal hearing underwent examination of their forearm flexor muscles (right and left sides; 20 samples in total) for VEMP acquisition. To this end, 200 tone burst stimuli at a 500 Hz frequency and 95 dBnHL intensity were promediated.

**RESULTS:**

No statistical differences were observed in VEMP responses acquired from the right and left forearm flexor muscles concerning P34 and N44 latencies (*p*=0.32 and 0.90, respectively). The mean latency obtained for the P34 wave component was 34.9 ms (±2.6), with a lower limit equal to 29.3 and an upper limit equal to 40.4 ms. The average latency of the N44 wave component was 43.6 ms (±2.1), with a lower limit of 39.1 ms and an upper limit of 48.1 ms. The results were consistent and had low variability, and showed an average asymmetry index of 15.4 (±10.7). These findings indicate that potentials may be investigated in different age groups and in specific clinical populations, such as pathologies that may alter the neuronal transmission of the inferior vestibular pathway, especially when a longer portion is observed.

**CONCLUSIONS:**

VEMP recording from forearm flexors is both feasible and stable, with latency reference ranges between 29.3 and 40.4 ms for P34, and 39.1 and 48.1 ms for N44.

## INTRODUCTION

Vestibular evoked myogenic potential (VEMP) is a muscular reflex resulting from strong auditory stimulation that depends on the integrity of structures such as the vestibulospinal pathway and the effector muscles (1,4).

VEMP has positive and negative components, which vary according to the capture location. The values corresponding to the contractile neck muscles - named cervical VEMP and the extraocular muscles-ocular VEMP - have been described previously in the literature, as well as their respective recording techniques (13,14).

Some studies refer to the possibility of VEMP capture from the upper limb muscles, such as the triceps; however, this is complex, and requires a large amount of stimuli. This modality is useful for evaluating lesions in the spinal cord anterior funiculus and, consequently, from the vestibulospinal path (15-19).

It is known that the vestibulospinal pathway is one of the main pathways that influences motor neuron excitability. Indeed, interruption of this central pathway results in a marked decrease in the tonic vibration reflex, excitability of motor neurons, and the anti-gravity muscle tone (5). In this sense, some diseases present pathway impairment, an example being the motor neuron diseases (MND), among which, amyotrophic lateral sclerosis (ALS) is the most common. Currently, the diagnosis for ALS is clinical, and supported by a physiological study performed through electroneuromyography, a high-cost examination that is complex in both its performance and interpretation (6-12).

In this context, and based on the clinical manifestations and affected pathways of ALS, it is believed that VEMP is able to identify functional alterations in the initial stages of the disease, since its main purpose is to analyze the degree of functioning and integrity of the physiological pathways of neurons.

A recent study demonstrated alterations in the cervical VEMP of patients with ALS; these patients had delays in P13 and N23 latencies when compared to the values found in healthy individuals (25). Due to the disease progression pattern of ALS, which starts in the distal limb areas, it is believed that the alterations in VEMP are greater in the upper limb extremity muscles, such as the brachioradialis and some forearm flexors, than those of the cervical VEMP.

Thus, the present study aimed to determine the measures of the central tendency and variability of VEMP with respect to the latency and wave amplitude, in addition to the asymmetry indexes between the right and left forearms, when captured from flexor muscles in healthy subjects. Our findings could serve as control parameters for further studies of specific clinical populations. It was also necessary to test the hypothesis that the observation of a longer portion of the inferior vestibular nerve results in more evident clinical findings for the diagnosis in question.

## MATERIAL AND METHODS

This research is part of a study to develop a new diagnostic test for MND. Initially, tests were carried out in a pilot group in order to adjust the analysis procedures. This group was composed of five volunteers, regardless of sex, aged between 18 and 40 years, with no history of hearing disorders. The muscles tested in this group were the brachioradialis and forearm flexors (radial carpal flexor and long palmar); the VEMP response obtained from the brachioradialis muscle in the pilot study was found to be less consistent than that acquired in the forearm flexors; thus, this muscle group was chosen to be studied in this research. The study was started following a few adjustments to the data collection instruments and technique.

A total of 20 samples, 10 from the right forearm and 10 from the left, were included in this study. Ten individuals, aged between 20 and 55 years, and of both sexes were included. Participants showed hearing thresholds ≤15 dBHL, with frequency differences between the right and left ears ≤10 dB. The exclusion criteria were as follows: exposure to occupational or leisure noise; ear surgery; more than three ear infections in the current year; use of ototoxic and/or psychotropic medication; presence of cochlear-vestibular problems; presence, or family history of neurodegenerative disease; and hormonal changes.

After reading and signing the free and informed consent form, participants were asked to respond to a questionnaire concerning their general health history and auditory and vestibular function, for volunteer screening. Otoscopy, pure tone audiometry. and VEMP procedures were performed soon after.

**Figure 1:**
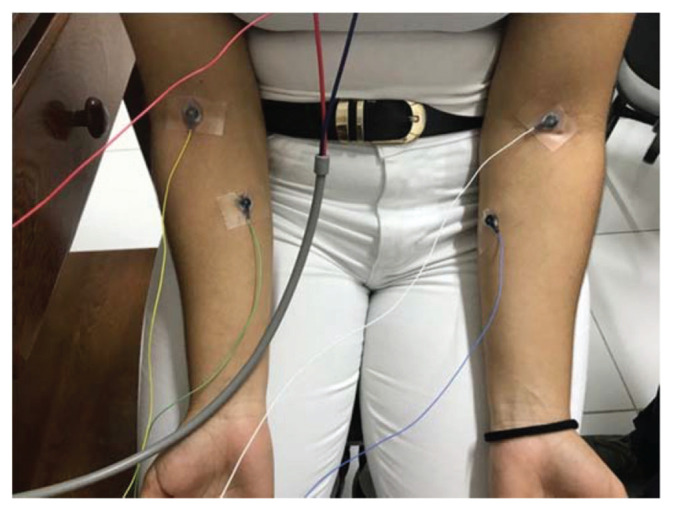
Positioning of electrodes on the forearm.

The Bio-logic Navigator PRO AEP system was used to obtain a VEMP signal from volunteers. Recordings were performed using disk-type electrodes placed on the participant’s skin. Initially, both forearms of each participant were palpated in search of the muscle group formed by the radial carpal flexor and the long palmar, on which the active electrode was placed. The reference electrode was positioned on the muscle group insertion, and the ground electrode was placed on the sternal manubrium (Figure 1).

Prior to recording, volunteers were asked to perform wrist flexion in order to contract the studied muscles. Then, 200 tone burst stimuli at 500 Hz frequency were promediated, with a Blackman ramp, plateau of one cycle and rise/fall of two cycles, and rarefaction polarity with intensity of 95 dBnHL (101 dBSPL in 500 Hz). The sound stimuli were presented by means of ER A3 insertion headphones, starting from the right afference and followed by the left. Responses were recorded three times on the right side and three times on the left.

Volunteers remained seated on a stretcher during the entire test. The VEMP responses were analyzed by two evaluators who analyzed the wave morphology, demarcating the P peak referring to the first major positive point, and N as the first major negative point. Then, the latencies and amplitudes of each point were recorded, and one wave was selected from each forearm side, for each volunteer.

The gold standard was established from the cervical VEMP captured from the sternocleidomastoid muscle, performed with the same parameters already described, to obtain the potentials captured in the forearm flexor muscles. The total time required to perform all the procedures in this study was 2 hours.

### Ethics

The study protocol is based on the CNS/MS 466/12 resolution for studies with human beings, and was approved by the Research Ethics Committee (no. 2.105.859). This is an observational and cross-sectional analytical study that was carried out at a university in Alagoas-Brazil. Our methodology also complies with the principles dictated by the Declaration of Helsinki.

### Statistical methods

Descriptive statistics techniques were applied, including tables and illustrative graphics. The data were tabulated and processed by SPSS version 23. Tabular and graphical presentation of the average, standard deviations, 95% confidence intervals, and hypothesis tests were used for data analysis.

The data obtained were characterized using descriptive statistics; the Kolmogorov-Smirnov adherence test was applied to assess the normality of the variable distributions. To compare the latencies and amplitudes of the P and N wave components between the right and left sides, the paired Student’s t-test was applied. Values were considered significant when *p*<0.05. Subsequently, the Kolmogorov-Smirnov test was applied again in order to analyze the normality of the latency distributions of each wave component, regardless of the right or left side. The normality limits for P and N latencies were then calculated using the central limit theorem applied to normal distributions to identify the lower and upper limits, which define at least 95% of normal cases for the new measure described in Equation 1.



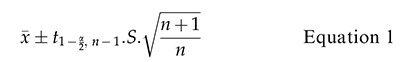



Where, x̵ is the sample mean, S is the sample standard deviation, n is the sample size (20), 1-α is the desired range (95%), t is the Student's t distribution percentile, and n-1 is the number of degrees of freedom (19).

Finally, normal average asymmetry indexes were calculated for the established amplitudes, as shown in Equation 2.



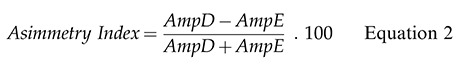



That is,



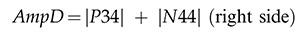





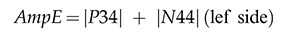



## RESULTS

The data obtained from the VEMP of 10 volunteers (20 samples, 10 from the right forearm and 10 from the left) were analyzed; among whom, five were male and five were female, and whose average age was 22 years.

The normality of the samples, regardless of the ear, was tested using the Kolmogorov-Smirnov test. All the studied variables showed a normal distribution, with *p*-values=0.20.

**Figure 2:**
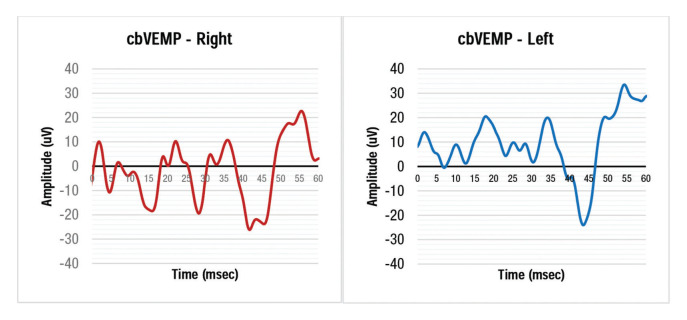
VEMP grand average of the right and left forearms.

The grand average of the VEMP waves of the forearm flexors obtained from the right and left sides is shown below (Figure 2).

With regards to the latencies P34 and N44, the data showed small numerical differences between the right and left sides, and the Student's t-test showed no significant difference (*p*=0.322 and 0.908, respectively). Thus, the acquired data may be arranged in a single group, regardless of right or left afference.

**Figure 3:**
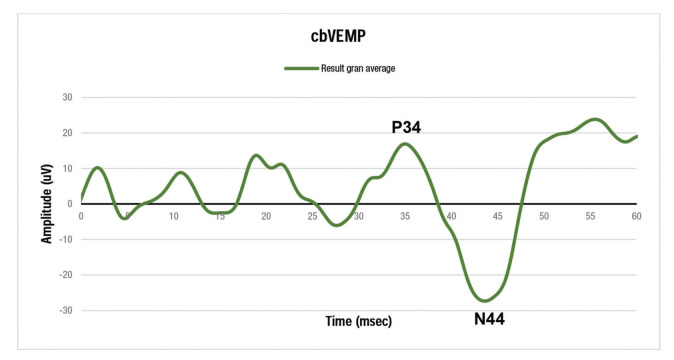
Grand average resulting from all VEMP examinations captured on the forearm, with emphasis on the P34 positive peak and the N44 negative peak.

The grand average of VEMP captured from the forearm, with emphasis on the P34 and N44, is shown in Figure 3.

The study of latencies and resulting amplitudes, averages and standard deviations, as well as the lower and upper limits which define at least 95% of normal cases for the measure described in the present study, are presented in Table 1. The mean latency obtained for the P34 wave component was 34.9 ms (± 2.6), with a lower limit equal to 29.3 and an upper limit equal to 40.4 ms. The average latency of the N44 wave component was 43.6 ms (± 2.1), with a lower limit of 39.1 and an upper limit of 48.1 ms. The N44-P34 interpeak interval was 8.7 ms, and the normal response was between 5.4 and 11.9 ms (Table 1).

Finally, asymmetry indexes for amplitudes between the right and left sides were calculated using the data shown in Table 2. The average asymmetry index for the amplitude between sides was 15.4% (±10.7; CI 95% 7.8%-23.1%).

## DISCUSSION

In the current study, for the first time, we described the acquisition of VEMP data from forearm muscles. In view of the novelty of this technique, some problems were found. Initially, data were acquired from the brachioradialis muscle, and from a group composed of forearm flexors, radial carpal flexor and long palmar. However, the responses of the brachioradialis muscle did not show good reproducibility; it is believed that other muscles may have interfered on the VEMP capture. Moreover, since brachioradialis participates in several movements, including flexion, pronation, and supination, individual and correct contraction is difficult to perform (20).

Data acquired from the radial carpal flexor and the long palmar showed good reproducibility in terms of responses, probably due to their synergistic performance in wrist flexion. In addition, the palpation technique was used to determine the location for the active electrode, due to anatomical differences inherent to each individual, which facilitated stimulus capture.

It is difficult to establish the exact electrode positioning since the forearm anterior face is composed of several small muscles, which may have led to variations in responses among individuals. In addition, differences in muscle strength may have also contributed to such variations (20,21).

Only one previous study that performed upper limb VEMP acquisition was found in the literature. In this work, the VEMP was obtained from the triceps muscle, however, considering the method used for data collection was associated with the form of muscle contraction, this was found to be complex, as well as not being feasible for ALS patients to perform, since they show motor limitations (22).

In the present study, participants were requested to perform wrist flexion in order to promote the contraction of the forearm flexors. This proved to be a simple alternative for upper limb VEMP capturing and was found to be feasible for patients with ALS or other neurological disorders.

The latency values obtained in this work were higher than those described for VEMPs in previous studies (23). Such differences may be justified by the distance between the studied muscles and the vestibulospinal pathway. This is reinforced by the values obtained for the P13 and N23 of triceps muscle by Cherchi et al. (22) (36.83±8.42 ms and 43.74±8.80 ms).

The standard deviation between the latency values was low in this study, and the asymmetry index was 15.4% (±10.7), which is considered normal, since the literature recommends asymmetries lower than 34% for VEMP captured in the sternocleidomastoid muscle (24).

Finally, our results were shown to be consistent, reproducible, and have low variability in latencies among the samples studied. We believe that this technique may be utilized in clinical investigations which will have the advantage of being able to observe a longer portion of the inferior vestibular branch. This measurement location may benefit the diagnosis of several diseases that affect the vestibulospinal pathway and the upper limbs, and it may demonstrate better results than the traditional exam (cervical VEMP) for some patient groups.

## CONCLUSIONS

VEMP obtained from the forearm flexors is viable and stable, with average latency values of 34.9±2.6 and 43.6±2.1 ms for P34 and N44, respectively.

## AUTHOR CONTRIBUTIONS

Valente MC contributed in project development, data collection, and writing of the manuscript. Carnauba AT and Andrade KC contributed in data collection and writing of the manuscript. Pai JD contributed in writing and translation of the manuscript. Menezes PL contributed in Guided the development of the research project, data collection, and writing of the manuscript.

## Figures and Tables

**Table 1:** Latencies, interpeak intervals, and resulting average amplitudes, standard deviations, and normal ranges for VEMP of the forearm.

Wave component	Average (ms)	Standard deviation	Inferior normal limit (CI 95% IL-SL)	Superior normal limit (CI 95% IL-SL)
P34 latency	34.9	2.6	29.3 (27.7-30.9)	40.4 (38.8-42.1)
N44 latency	43.6	2.1	39.1 (37.7-40.4)	48.1 (46.7-49.4)
N44-P34 interval	8.7	1.5	5.4 (4.5-6.4)	11.9 (10.9-12.8)
P34 amplitude	43.3	20.8	-	-
N44 amplitude	-36.9	30.0	-	-

Note: The limits of normality for amplitudes were not calculated since the asymmetry index was used for this measurement.

**Table 2:** Average amplitudes, standard deviation, and asymmetry index between the ears for the forearm VEMP.

Wave component	Average (ms)	Standard deviation	% Average asymmetry index (SD)
P34 amplitude (R)	38.0	16.4	15.4 (±10.7)
N44 amplitude (R)	-33.8	18.6	
P34 amplitude (L)	48.6	25.1	
N44 amplitude (L)	-40.0	40.7	

R: Right side, L: Left Side.
